# Molecular phylogeny and taxonomy of the genus *Vernaya* (Mammalia: Rodentia: Muridae) with the description of two new species

**DOI:** 10.1002/ece3.10628

**Published:** 2023-11-09

**Authors:** Songping Zhao, Xuming Wang, Binbin V. Li, Liang Dou, Yingxun Liu, Siyu Yang, Ronghui Fan, Yong Jiang, Quan Li, Rui Liao, Miao Hu, Xuelong Jiang, Shaoying Liu, Shunde Chen

**Affiliations:** ^1^ Ministry of Education Key Laboratory of Land Resources Evaluation and Monitoring in Southwest (Sichuan Normal University) Chengdu China; ^2^ Sichuan Normal University Chengdu China; ^3^ Sichuan Academy of Forestry Chengdu China; ^4^ Environment Research Center Duke Kunshan University Kunshan China; ^5^ Nicholas School of the Environment Duke University Durham North Carolina USA; ^6^ Sichuan University Chengdu China; ^7^ Gongga Mountain National Nature Reserve Administration of Sichuan Province Kangding China; ^8^ State Key Laboratory of Genetic Resources and Evolution and Yunnan Key Laboratory of Biodiversity and Ecological Conservation of Gaoligong Mountain, Kunming Institute of Zoology The Chinese Academy of Science Kunming China

**Keywords:** morphometric, new species, taxonomy, *Vernaya*

## Abstract

The climbing mouse is a rare, small mammal listed as an endangered species on the China species red list. Molecular phylogenetic analyses and the evolutionary history of the genus remain unexplored because of the extreme difficulty in capturing individuals and their narrow distribution. Here, we collected 44 specimens, sequenced one mitochondrial and eight nuclear genes, and integrated morphological approaches to estimate phylogenetic relationships, delimit species boundaries, and explore evolutionary history. Molecular analyses and morphological results supported the validity of these four species. Here, we describe two new species, *Vernaya meiguites* sp. nov. and *Vernaya nushanensis* sp. nov., and recognize *Vernaya foramena*, previously considered a subspecies of *Vernaya fulva*, as a valid species. The estimated divergence time suggests that the climbing mouse began to diversify during the Pliocene (3.36 Ma).

## Graphical abstract

The climbing mouse (genus *Vernaya*) is one of the least studied small mammals. This manuscript integrates molecular and morphological approaches to study the phylogeny and taxonomy of the genus. They described two new species and recognized *Vernaya foramena* as a valid species. It makes a potentially important contribution to our understanding of the systematics of the genus *Vernaya*.

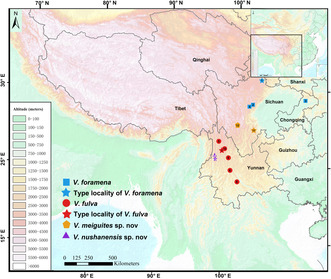

## 1 INTRODUCTION

Southwestern China has an extremely unique and complex topographic structure with high species richness. He and Jiang (2014) detailed the phylogeographic structure of sky islands in southwestern China, revealing the factors influencing species evolution and the general pattern of allopatric speciation. The number of species in China has markedly increased in recent years, mainly because of the discovery of new species (Jiang et al., 2015; Wilson & Reeder, 2005). New species of small mammals have been frequently reported in southwestern China (Chen et al., 2017; Liu et al., 2019; Pu et al., 2022), indicating that species diversity remains underestimated. Some unsystematically studied species require further classification and revision (Chen et al., 2017).

*Vernaya* is a monotypic genus belonging to the subfamily Murinae of the family Muridae, in the order Rodentia (Musser, 1979; Musser & Carleton, 2005). It is distributed in the mountainous areas of southwestern China (western Yunnan, central and northern Sichuan, southern Gansu, and southwestern Shaanxi) and extends to northern Myanmar (Allen, 1927; Anthony, 1941; Li & Wang, 1995; Musser & Carleton, 2005; Smith et al., 2008; Wang et al., 1980). *Vernaya fulva* was first collected in Lanping, Yunnan, in 1916 by Andrews and Heller. In 1927, Allen characterized it as a member of the genus *Chiropodomys* and named it *Chiropodomys fulvus*. After several revisions, it was finally named *Vernaya*, and the species name was changed from *fulvus* to *fulva* (Allen, 1927; Anthony, 1941; Lunde, 2007). A group of specimens from Wanglang, Sichuan, was identified as the new species, *Vernaya foramena* (Wang et al., 1980). Subsequently, Li and Wang (1995) considered *V. foramena* to be a geographic subspecies of *V. fulva* (Li & Wang, 1995), namely *V. f. foramena*. This arrangement has been followed in many subsequent studies (Lunde, 2007; Smith et al., 2008; Wang, 2003; Wei et al., 2021; Wilson et al., 2018). In addition, the remnants of *V. fulva* and four extinct species from the same genus were found in late Pleistocene cave sediments in the Sichuan–Guizhou region of southern China (Zheng, 1993). *Vernaya fulva* is the only extant species in the genus and is listed as endangered (EN) on the China species red list (Jiang et al., 2021). According to the International Union for Conservation of Nature (IUCN) Red List Categories (Aplin, 2017), *Vernaya fulva* belongs to the category of “Least Concern.”

However, the molecular systematics and evolutionary history of this genus have not yet been studied. Whether *V. f. foramena* can be considered an independent, valid species has not yet been analyzed from a systematic molecular perspective. In this study, we collected 44 specimens of the genus *Vernaya* from Sichuan, Chongqing, and Yunnan provinces in China. Morphological characteristics, principal component analysis of skulls, and mitochondrial and nuclear genes were used to (1) determine whether *V. f. foramena* is an independent valid species, (2) infer the phylogenetic relationships of the genus *Vernaya*, and (3) explore the evolutionary history of the genus *Vernaya*.

## 2 MATERIALS AND METHODS

### 2.1 Molecular data and analyses

**FIGURE 1:**
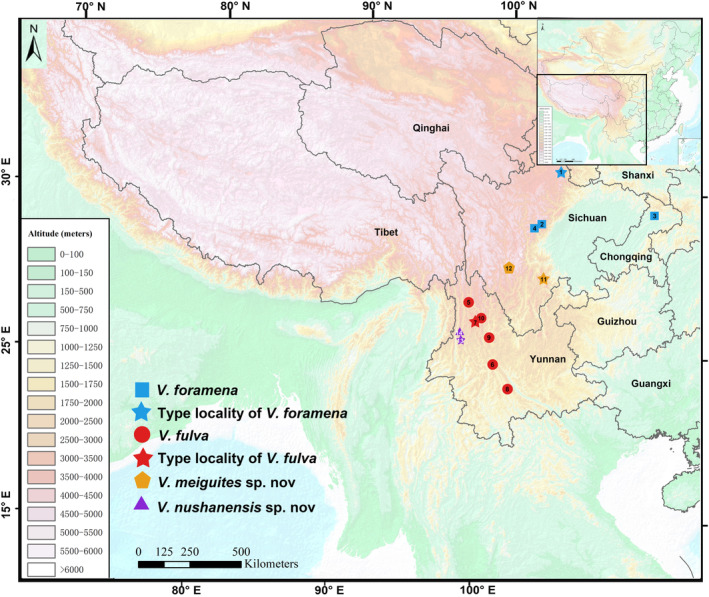
Map of the collection sites of the genus *Vernaya* in this study.

From 1979 to 2022, 44 *Vernaya* specimens from Sichuan, Chongqing, and Yunnan provinces in China were collected using snap traps (Figure 1 and Table 1). All samples were numbered, and their morphological data [including head‐body length (HBL), tail length (TL), ear length (EL), and hind foot length (HFL)] were measured. Fresh liver or muscle tissues were collected from 20 individuals and stored in 99% pure analytical ethanol. After returning to the laboratory, the tissue samples were stored at −80°C after changing the analytical alcohol for molecular studies. All individuals were made into the study skins. Tissues and specimens used in this study were stored at the Sichuan Academy of Forestry (SAF), College of Life Sciences of Sichuan Normal University (SCNU), Kunming Institute of Zoology (KIZ), and College of Life Sciences of Sichuan University (SCU) (Table 1).

**TABLE 1:** Information on the collection sites of *Vernaya* specimens in this study.

	Site	Field ID	Museum number	Locality	Longitude	Latitude	Elevation (m)
*V. foramena*	1	CSD2275	SAF181656	Pingwu, Sichuan	104.054925	32.986575	2700
	2	CSD2380	SAF14291	Wenchuan, Sichuan	102.98167	30.88806	3000
	3	CSD2381	SAF16726	Kaizhou, Chongqing	108.709593	31.456538	1218
	4	CSD2382	SAF08388	Baoxing, Sichuan	102.72578	30.80798	2450
	4	CSD2383	SAF08490	Baoxing, Sichuan	102.7531	30.84053	2680
	1	CSD3538	SAF201560	Pingwu, Sichuan	104.0175	33.00222	2812
	1	CSD3539	SAF201553	Pingwu, Sichuan	104.0175	33.00222	2812
	1	CSD3540	SAF201518	Pingwu, Sichuan	104.02015	33.00484	2921
	1	CSD3541	SAF201470	Pingwu, Sichuan	104.02015	33.00484	2921
	1	CSD3566	SAF20233	Pingwu, Sichuan	104.019241	32.995995	2880
*V. fulva*	5	CSD2384	SAF15422	Weixi, Yunnan	99.38623	27.21222	3250
	6	CSD2410	SAF18802	Jingdong, Yunnan	101.0303	24.54656	2493
	6	CSD2411	SAF18803	Jingdong, Yunnan	101.0303	24.54656	2493
	7	CSD4405	SCNU02747	Lanping, Yunnan	99.371662	26.344607	2245.29
	6	97,339	KIZ:018995	Jingdong, Yunnan	–	–	2860
	6	811,532	KIZ:014782	Jingdong, Yunnan	–	–	2460
	6	811,531	KIZ:014781	Jingdong, Yunnan	–	–	2460
	6	811,528	KIZ:014778	Jingdong, Yunnan	–	–	2460
	8	811,537	KIZ:014787	Xinping, Yunnan	–	–	–
	8	811,538	KIZ:014788	Xinping, Yunnan	–	–	–
	5	830,270	KIZ:007619	Weishan, Yunan	–	–	–
	10	79,868	KIZ:005009	Jianchuan, Yunnan	–	–	2800
	6	811,530	KIZ:014780	Jingdong, Yunnan	–	–	2460
	6	811,533	KIZ:014783	Jingdong, Yunnan	–	–	2460
	6	811,534	KIZ:014784	Jingdong, Yunnan	–	–	2460
	6	811,535	KIZ:014785	Jingdong, Yunnan	–	–	2460
	6	811,536	KIZ:014786	Jingdong, Yunnan	–	–	2460
	6	811,533	KIZ:014783	Jingdong, Yunnan	–	–	2460
	6	811,529	KIZ:014779	Jingdong, Yunnan	–	–	2460
*V.* sp1	11	CSD3536	SAF201652	Meigu, Sichuan	103.051	28.64858	2616
	11	CSD3537	SAF201653	Meigu, Sichuan	103.051	28.64858	2616
	12	CSD6130	SAF220266	Jiulong, Sichuan	101.523272	29.124803	3300
	11	–	MCU104071	Meigu, Sichuan	–	–	–
	11	–	MCU104072	Meigu, Sichuan	–	–	–
	11	–	MCU104073	Meigu, Sichuan	–	–	–
	11	–	MCU104074	Meigu, Sichuan	–	–	–
	11	H′‐3‐10	MCU104075	Meigu, Sichuan	–	–	–
	11	D‐5‐4	MCU104076	Meigu, Sichuan	–	–	–
	11	–	MCU104077	Meigu, Sichuan	–	–	–
	12	–	MCU104078	Meigu, Sichuan	–	–	–
*V.* sp2	13	CSD3561	SAF19287	Yunlong, Yunnan	99.11484	25.75724	2500
	14	XMS170200	KIZ:XMS170200	Lushui, Yunnan	98.93872	25.96119	3470
	14	XMS170115	KIZ:XMS170115	Lushui, Yunnan	98.94525	25.95947	3575

Abbreviations: KIZ, Kunming Institute of Zoology; SAF, Sichuan Academy of Forestry; SCNU, Sichuan Normal University; SCU, College of Life Sciences of Sichuan University.

Total DNA was extracted using an Animal Tissue Genomic DNA Rapid Extraction Kit (Chengdu Fuji Biotechnology Co., Ltd.). The primers used for PCR amplification are shown in the Table S1. PCR conditions consisted of an initial denaturing step at 94°C for 5 min, followed by 35 cycles of 45 s denaturation at 94°C, 45 s annealing at 49–60°C, 90 s extension at 72°C, and a final extension step at 72°C for 10 min.

Tissue samples from 20 climbing mice were amplified, sequenced, and analyzed. We sequenced one mitochondrial gene (CYTB, 1140 bp) for all samples and eight nuclear genes (recombination activating 1 [RAG1, 1080 bp]), including inverted‐repeat‐binding protein [IRBP, 1142 bp], growth hormone receptor [GHR, 748 bp], adenosine A3 receptor [ADORA3, 342 bp], administered beta 2 [ADRB2, 802 bp], amyloid beta precursor protein [APP, 296 bp], adenosine triphosphate 7a [ATP7A, 604 bp], and brain‐derived neurotrophic factor [BDNF, 561 bp], which are good genetic markers for determining the phylogenetic relationships of some mammals (He et al., 2017; Meredith et al., 2011). Sequencing was performed by Beijing Tsingke Biotech Co. Ltd. The original sequences were obtained via comparison using Mega 5.0 (Tamura et al., 2011), supplemented by manual correction, splicing, and confirmation of the normal translation of all sequences. The sequence data of the outgroups (*Mastomys coucha*, *Mus musculus*, and *Rattus norvegicus*) were downloaded from GenBank (Table S2).

Phylogenetic analyses were conducted using the following three datasets: a mitochondrial dataset (CYTB), an all‐gene combined dataset (mtDNA + nuDNA), and an all‐nuclear gene dataset (nuDNA). We used jModelTest 2.1.7 (Darriba et al., 2012) to detect the optimal model for each gene. The fitness of the model was evaluated using the Akaike Information Criterion (Luo et al., 2010), and the best model for each gene is shown in Table S1.

Bayesian tree reconstruction was performed using BEAST v1.7 (Drummond et al., 2012), and each analysis consisted of 100 million generations, which were sampled every 5000th generation. The relaxed uncorrelated lognormal clock model and Yule species formation model were used for each operation. Statistics were performed using Tracer v1.6 (Rambaut & Drummond, 2013) to confirm that the effective sample sizes (ESSs) were greater than 200. The first 10% of the trees were discarded as burn–in using TreeAnnotator v1.6.1. We used Figtree v1.4.3 (Rambaut, 2016) to perform the output of the phylogenetic tree, and posterior probabilities (PP) >.95 were considered to be strongly supported (Huelsenbeck & Rannala, 2004).

Genetic distances were calculated based on the CYTB gene using the Kimura two–Parameter (K2P) model (Kimura, 1980) in Mega 5.0 (Tamura et al., 2011), with 1000 bootstrap replications.

Species were identified using a monothreshold GMYC model based on a maximum genealogical confidence tree of mitochondrial genes (Pons et al., 2006). The GMYC analysis was performed using the time‐calibrated gene tree as the input tree derived from the mitochondrial dataset without outgroups, and the split package was applied to the R environment for species delimitation (Paradis et al., 2004).

To verify the genetic differentiation detected in the genus *Vernaya*, the gene tree constructed in the previous step was used as a guide tree, and BPP v3.1 (Yang & Rannala, 2010) was used to test the independent evolutionary lineage hypothesis of these populations under multi‐species fusion (Yang & Rannala, 2010). Datasets 2 (mtDNA + nuDNA) and 3 (nuDNA) were included in the analysis. Algorithms 0 and 1 were used to specify the rjMCMC moves between the different species‐partitioning models. We set the size and divergence time of the ancestral population using two prior settings: (1) a relatively large ancestral population and shallow branches [G (1, 10) for θ and G (2, 2000) for τ]; and (2) a relatively small ancestral population and shallow branches [G (2, 2000) for θ and G (2, 2000) for τ]. Each rjMCMC was run for 100,000 generations and sampled for each 100a generation after discarding 10,000 generations as pre burn‐in. If the posterior probability was greater than .95, each clade was considered an independent species.

Divergence time was estimated on the nuDNA dataset, which was analyzed in BEAST v1.7 (Drummond et al., 2012). Two fossil calibration points were used to evaluate the divergence time for the group. All fossil calibration age constraints were treated as log‐normal distributions (Ho, 2007). (1) The split of *Mus* and *Rattus* was approximately 15.9 Ma (upper 95%, 15.9–16.41 Ma) (He et al., 2019). (2) The first fossil record of the genus *Mus auctor* (Jacobs & Pilbeam, 1980; Lundrigan et al., 2002) indicated a minimum difference of 5.7 Ma (upper 95%, 5.36–6.21 Ma) between different house mouse lineages. The BEAST analysis used a Yule tree prior and a relaxed lognormal clock model. Each analysis ran for 100 million generations and was sampled every 5000th generation. The posterior distribution and ESS of each parameter higher than 200 were calculated in Tracer v1.6 (Rambaut & Drummond, 2013). TreeAannotator v1.6.1, which was set to the first 10% of the generations, was used to determine the necessary burn‐in fraction.

### 2.2 Morphological data and analyses

The external measurement data for the specimens were obtained from the specimen labels. Skull measurements followed those of Liu et al. (2007, 2012) and Patton and Conroy (2017). A digital display vernier caliper (accurate to 0.01 mm) was used to measure 11 indices, including the greatest length of the skull (GLS), braincase height (BH), skull basal length (SGL), palatal length (PL), zygomatic breadth (ZB), upper toothrow length (UTL), upper molar row length (UML), breadth across upper molars (BUM), lower toothrow length (LTL), lower molar row length (LML), and mandibular length (ML). The specimens of the measured species of *Vernaya* are shown in the Table S3. The measured data were analyzed using SPSS (version 20.0) for correlation. The overall similarity between species was evaluated using principal component analysis (PCA), under the condition that all data satisfied a normal distribution. A discriminant analysis was performed to determine whether the classification of the specimens was accurate.

We recorded the morphological characteristics of the male genitalia (Hooper, 1958), prepared the glans penis using standard methods (Hooper, 1958; Lidicker, 1968), and characterized the bacula structures (Yang & Fang, 1988). The glans were preserved in 75% ethanol. Dissection was performed using a binocular microscope, and the morphologies of the urethral lappet, dorsal papilla, and outer crater papilla were observed and described. The bacula were prepared according to the established methods (Liu et al., 2007, 2012). Morphologies of the proximal, distal, and lateral baculae were observed under a binocular dissecting microscope.

## 3 RESULTS

### 3.1 Molecular results

**FIGURE 2:**
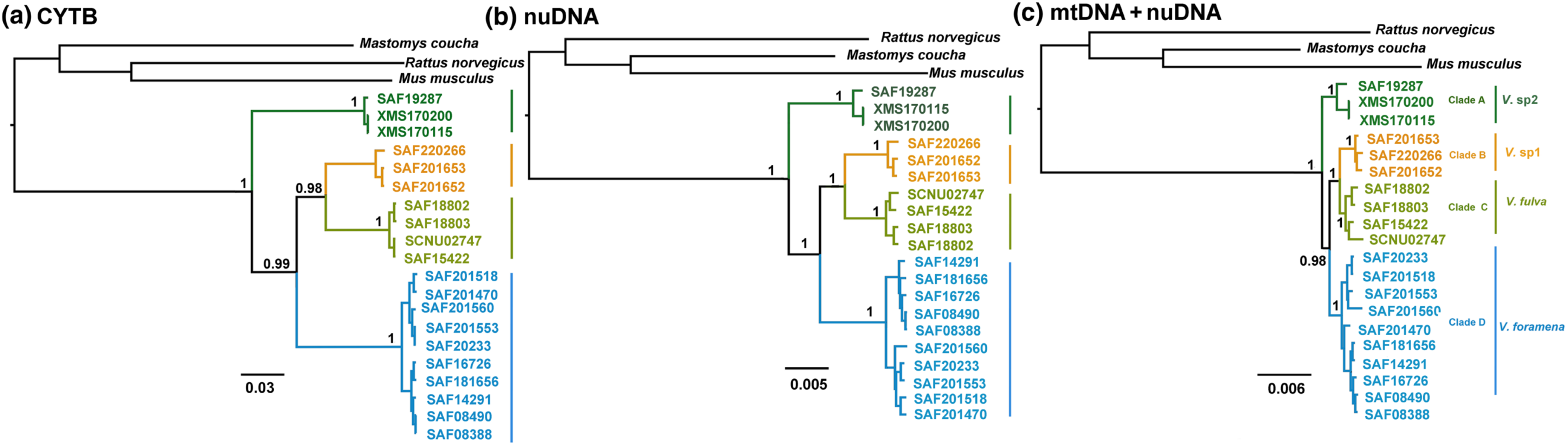
Bayesian phylogenetic analyses based on (a) dataset 1 (CYTB), (b) dataset 2 (eight nuclear genes concatenated, nuDNA), and (c) dataset 3 (CYTB and eight nuclear genes concatenated, mtDNA + nuDNA). The numbers above the branches refer to Bayesian posterior probabilities (PP).

CYTB for all individuals and nuclear genes for all individuals except the BDNF gene (two individuals) and ATP7A gene (three individuals) were obtained in this study, that is, 1140 bp for CYTB and 5575 bp for nuDNA. New sequence information was deposited in GenBank (Accession Numbers OR282742–OR282761, OR333541–OR333696, Table S2). BEAST was used to reconstruct phylogenetic relationships based on the three datasets, and the topologies obtained were approximately the same (Figure 2) (*Mastomys coucha*, *Mus musculus*, and *Rattus norvegicus* were used as outgroups). This topology strongly supports the division of the genus *Vernaya* into four clades. Clade A, representing *V*. sp2, was monophyletic and consistently placed as the most basal lineage in the three datasets (all pp = 1.0). *Vernaya* sp1 (clade B) was classified as the sister branch of *V. fulva* (clade C). The branches of clades B and C were sister groups to clade D of *V. foramena* (Figure 2).

**FIGURE 3:**
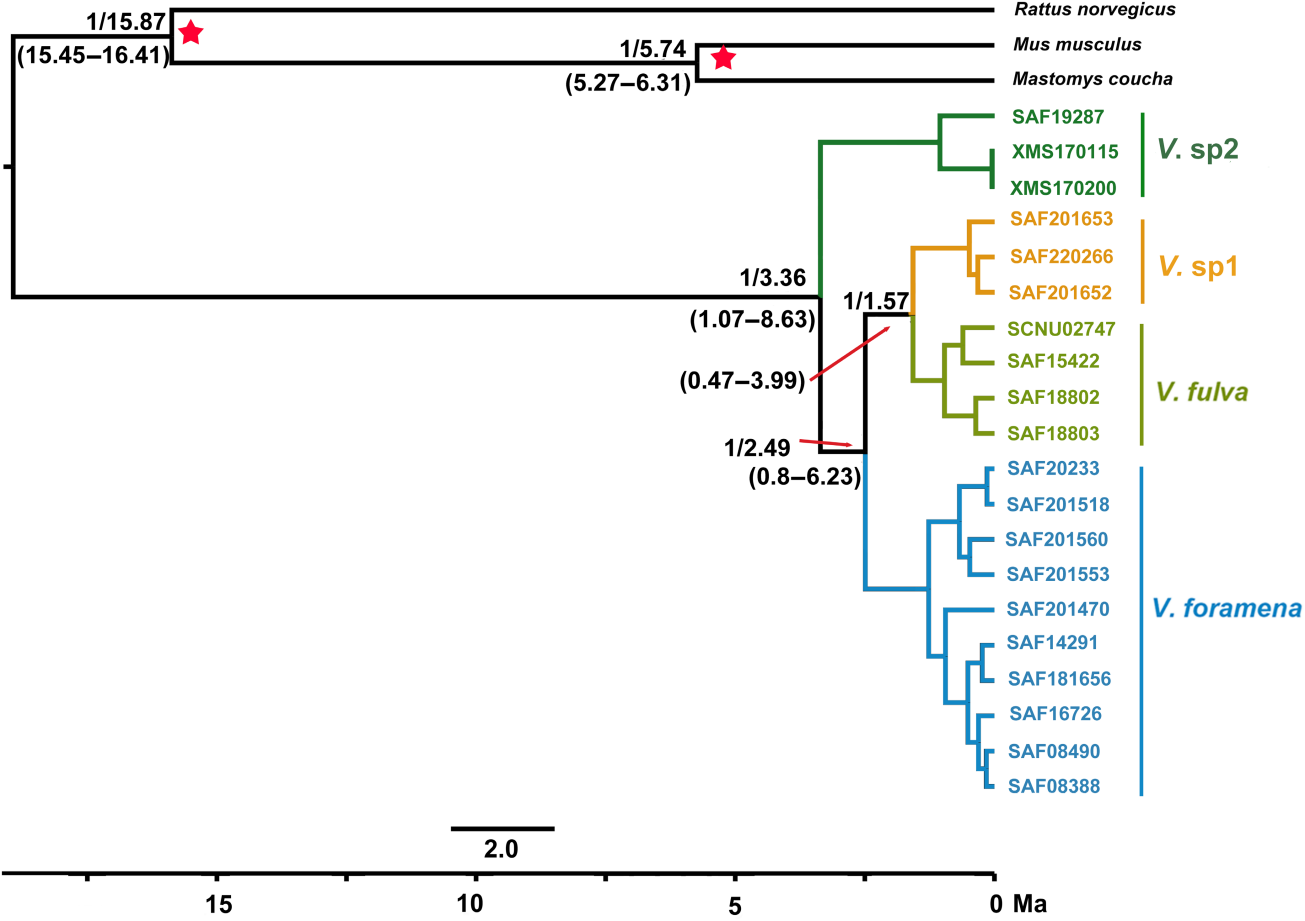
Divergence times were estimated using BEAST based on a nuDNA dataset. Branch lengths represent time. Numbers left of the dash indicate posterior probabilities (PP), numbers right of the dash represent the median divergence time, and numbers within parentheses indicate the confidence interval. The two red asterisks indicate fossil‐calibrated nodes.

According to BEAST v1.7, the topological structure of the divergence time tree was the same as that of the nuDNA gene tree. The results showed that the latest common ancestor of *Vernaya* can be traced back to the Pliocene (3.36 Ma, 95% CI = 1.07–8.63), which is also the time when *V*. sp2 diverged from other species. Both the divergence time of clade B and C (2.49 Ma, 95% CI = 0.8–6.23) and the divergence time of *V*. sp1 and *V. fulva* (1.57 Ma, 95% CI = 0.47–3.99) occur in the Pliocene (Figure 3).

The K2P genetic distance of the CYTB gene between the four putative *Vernaya* species was calculated. The results showed that the genetic distance ranged from 0.078 to 0.144 (Table 2). The genetic distance between *V*. sp2 and *V. foramena* was 14.4%, followed by 12.7% between *V*. sp2 and *V. fulva*. The minimum genetic distance occurred between *V. fulva* and *V*. sp1 and was found to be 7.8%. Genetic distances among all species showed good species‐level differences.

**TABLE 2:** Kimura two‐parameters (K2P) genetic distances in the genus *Vernaya* based on the CYTB gene.

	*V.* sp2	*V.* sp1	*V. foramena*
*V.* sp2			
*V.* sp1	0.114		
*V. foramena*	0.144	0.112	
*V. fulva*	0.127	0.078	0.116

The species delimitation results for the GMYC and BPP were identical. GMYC results divided the groups into four putative species (Figure S1). The BPP results are shown in Table S4, and the results of both datasets support four putative species.

### 3.2 Morphological results

**FIGURE 4:**
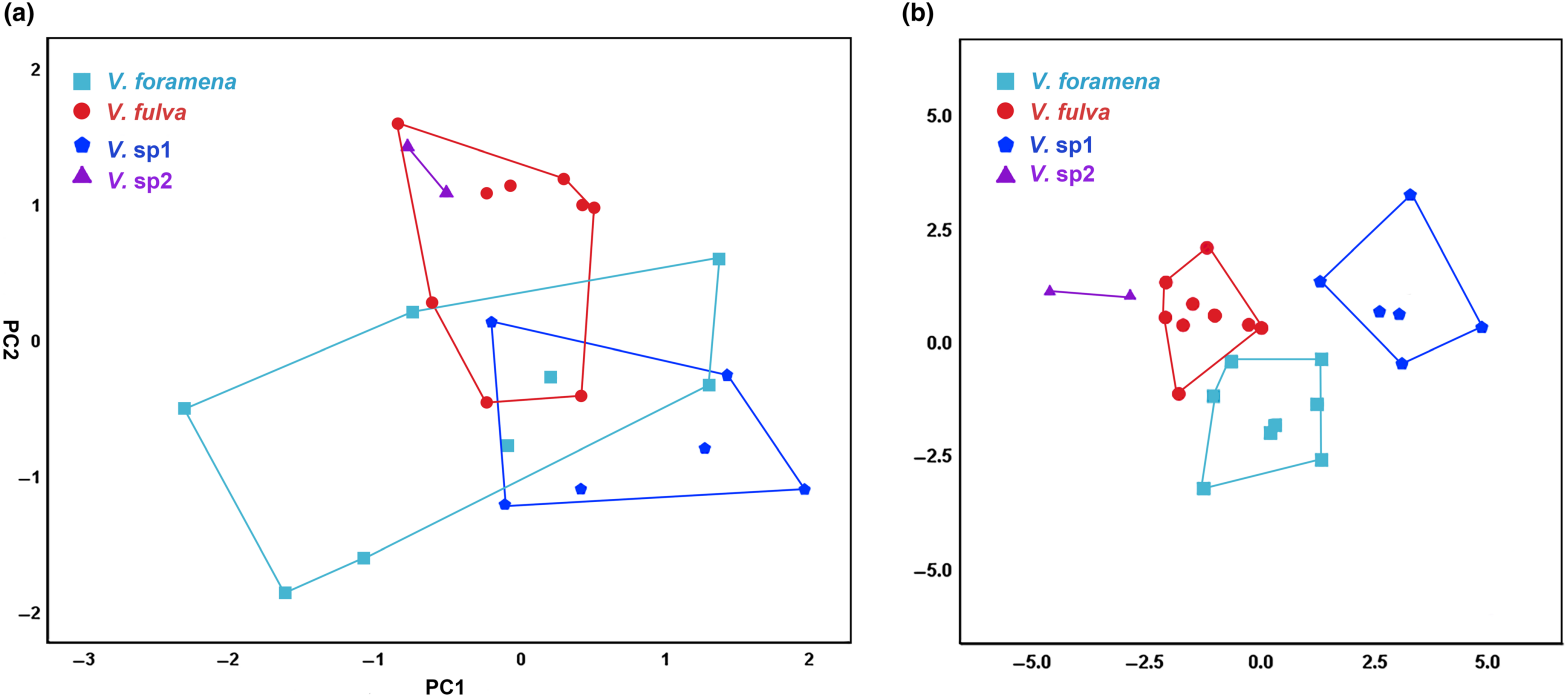
Principal component analysis (PCA) based on skull measurement data of genus *Vernaya* (a) results of PCA and (b) canonical discriminant analysis of *Vernaya*.

Eleven cranial measurements and four appearance traits were obtained for the four putative species (Table S3). The resulting value of the Kaiser–Meyer–Olkin measure of sampling adequacy (KMO) = 0.837 (Bartlett's test < 0.001) indicated that the data were suitable for PCA. The first two PCs, which explained 76.97% of the variance (Table 3), were selected to analyze *Vernaya*. The explanatory degree of PC1 was the highest at 59.87%, and all factor loadings were positive. The factor loadings of the UTL, PL, and SBL were >0.9. Factor loadings >0.8 included GLS and BUM (Table 3); the explanatory degree of PC2 was 17.10% and was negatively correlated with LTL, ML, LUM, and LLM (loadings <−0.2). All other loadings were >0.02 (Table 3). The scatterplots showed that some individuals of different species partially overlapped and still exhibited a good separation trend (Figure 4a). The variation in *V. foramena* was large, mainly distributed in the negative region of PC2 (Figure 4a), whereas most of *V. fulva* was distributed in the positive region, indicating that the skull was large and the braincase was wide. Most of *V*. sp1 occurs along the positive region of PC1 and the negative region of PC2 (Figure 4a), indicating that it has a relatively large skull and small braincase. *V*. sp2 was distributed in the positive region of PC2 and negative region of PC1 (Figure 4a), but only for reference owing to the small number of specimens.

**TABLE 3:** Factor loadings and percentage of variance explained for principal component analysis.

Variables	PC1	PC2
UTL	0.967	0.021
PL	0.918	0.107
SBL	0.914	0.128
GLS	0.892	0.266
BUM	0.841	0.243
LTL	0.770	−0.571
ML	0.717	−0.222
LUM	0.678	−0.571
LLM	0.637	−0.553
ZB	0.587	0.713
BH	0.390	0.515
Variance explained (%)	59.87	17.10

The results of the discriminant analysis showed that 92% of the specimens were accurately classified. Except for one individual of *V. foramena* that was assigned to *V. fulva* and one individual of *V. fulva* that was assigned to *V. foramena* (Table S5), all four putative species could be clearly distinguished (Figure 4b).

**FIGURE 5:**
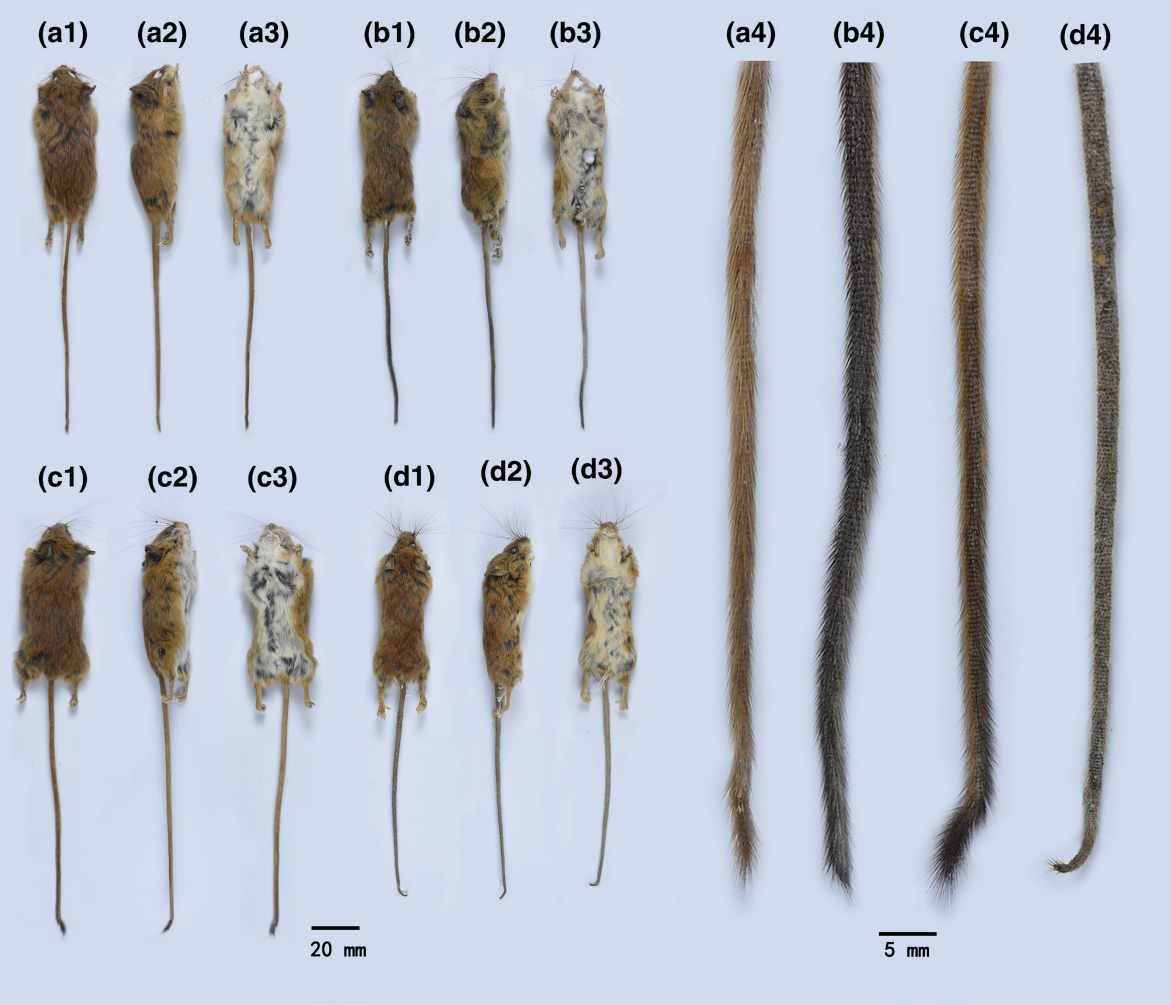
Pelage and tail comparisons among species of the genus *Vernaya*, (a) *Vernaya foramena* a1–a4 (museum number SAF201553); (b) *Vernaya fulva* b1–b4 (museum number SCNU02747); (c) *Vernaya* sp1 c1–c4 holotype (field number 2020MG045, museum number SAF201652); (d) *Vernaya* sp2 d1–d4 holotype (museum number SAF19287). (1) ventral view; (2) dorsal view; (3) lateral view; (4) tail variation.

**FIGURE 6:**
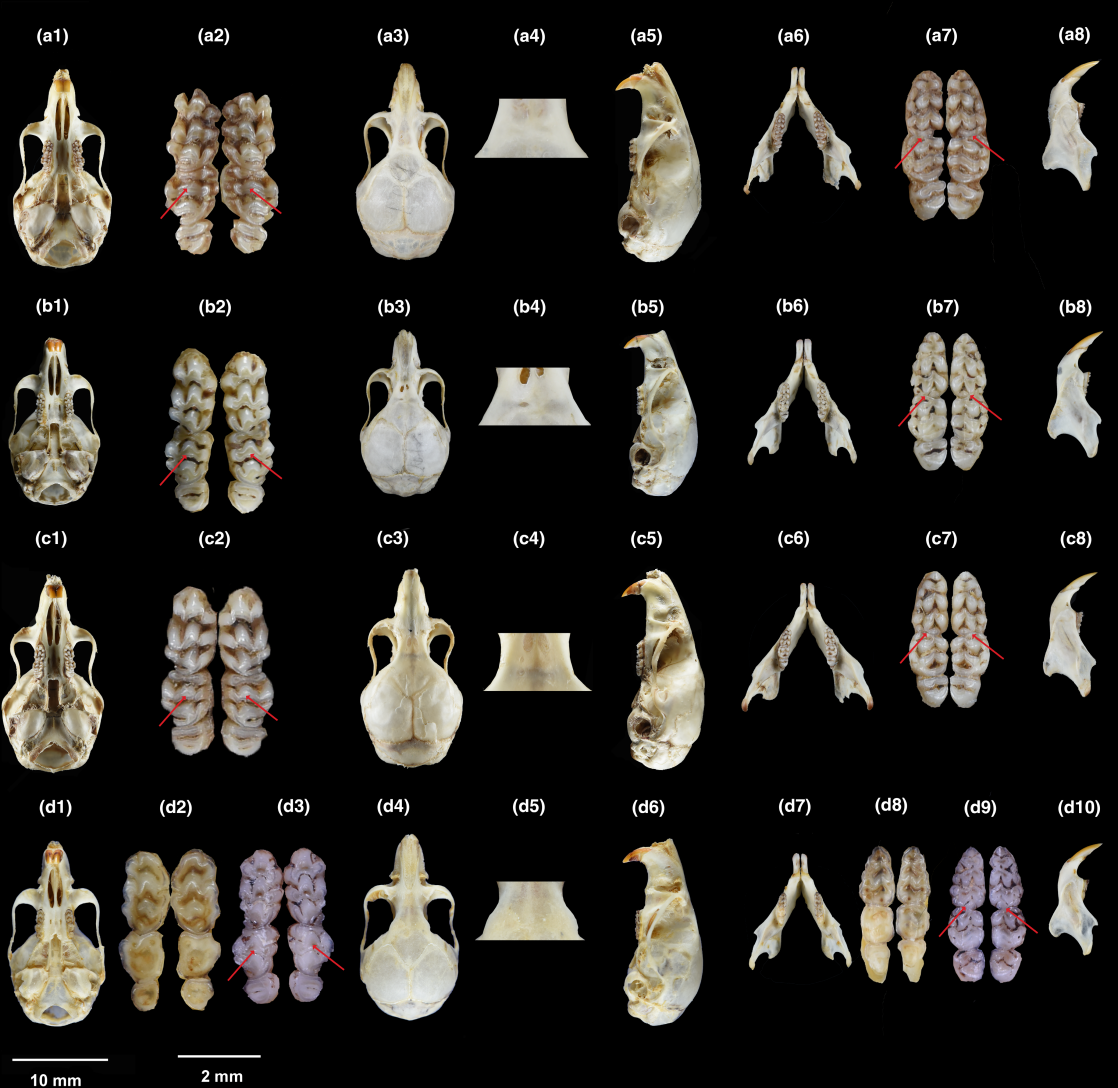
Skulls and tooth comparison among species of the genus *Vernaya*. (a) *Vernaya foramena* a1–a8 (museum number SAF201553); (b) *Vernaya fulva* b1–b8 (museum number SCNU02747); (c) *Vernaya* sp1 c1–c8 holotype (field number 2020MG045, museum number SAF201652); (d) *Vernaya* sp2 d1–d2, d4–d8, d10 holotype (museum number SAF19287), d3, d9 (museum number XMS170200). (1) Ventral view; (2, d3) upper tooth row; (3, d4) dorsal view; (4, d5) posterior orbital process; (5, d6) lateral view; (6, 8, d7, d10) lower jaws; (7, d8, d9) lower tooth row; The red arrow indicates the position of the dental variation.

**FIGURE 7:**
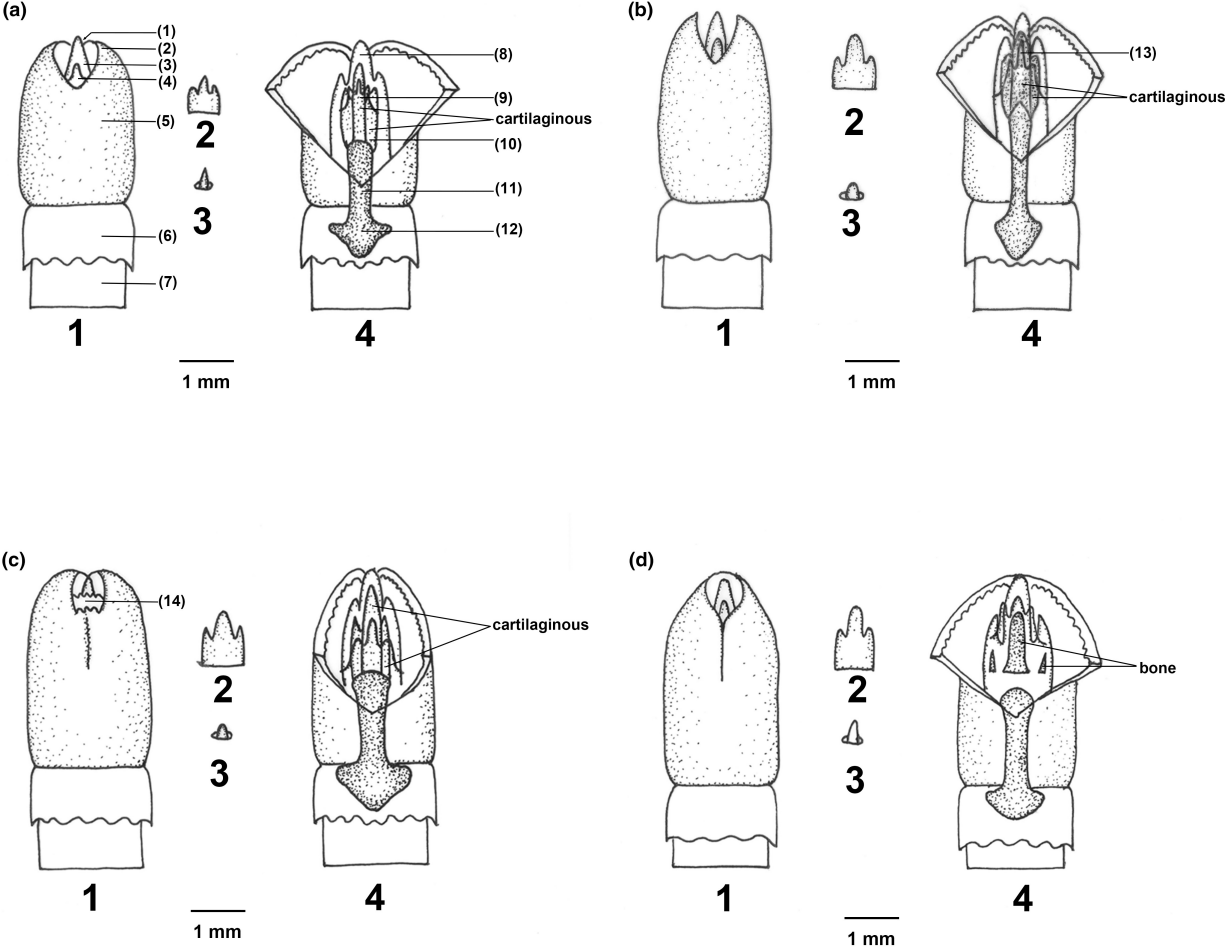
Penis comparison among species of the genus *Vernaya*. (a) *Vernaya foramena*. (b) *Vernaya fulva*. (c) *Vernaya* sp1. (d) *Vernaya* sp2. 1. Penis; 2. Urethral papilla; 3. Dorsal papilla; 4. Midventral cut view of glans. In (a–c), numbered structural features are as follows: (1) Site of dorsal papilla; (2) Outer crater; (3) Tip of distal baculum; (4) Tip of urethral papilla; (5) Glans; (6) Prepuce; (7) Body of penis; (8) Connective tissue enclosure of distal baculum; (9) Distal baculum; (1))Lateral baculum; (11) Proximal baculum; (12) The base of proximal baculum; (13) Urethral papillia; and (14) Inner crater.

Comparisons of the morphology and measurements among the different taxa of the four putative species are summarized in Tables 4 and 5, Table S3, and Figures 5 and 6. A morphological comparison of the penises is presented in Table 6 and Figure 7. The different species had distinct tail types (Figure 5). The tails of *V. foramena*, *V. fulva*, and *V*. sp1 are all covered with dense and long hairs (Figure 5a4,b4,c4), among which *V. foramena* has brownish yellow hairs and the dorsal and ventral uniformity (Figure 5a4); *V. fulva* has black hair and the ventral part is visibly lighter than the dorsal part (Figure 5b4); *V*. sp1, the hair on the back of the tail is yellow from the base to two‐thirds of the tail, black from one‐third of the tail, spindle‐shaped dense hair clusters formed at the tail, and yellow hair on the ventral (Figure 5c4). *V*. sp2 was covered with short and sparse hair and was gray (Figure 5d4).

**TABLE 4:** The average and standard deviation of the measurement data of the appearance and skull morphology of the specimens of the genus *Vernaya* used in this study.

Measurement	*V. foramena*	*V. fulva*	*V.* sp1	*V.* sp2
	*n* = 11	*n* = 17	*n* = 4	*n* = 3
Head body length	63.18 ± 5.84	67.71 ± 7.39	67.00 ± 7.53	71.33 ± 3.21
	54–73	54–80	58–69	69–75
Tail length	111.70 ± 8.33	124.88 ± 9.50	127.75 ± 3.10	124.67 ± 10.41
	100–125	112–143	125–132	113–133
Ear length	16.77 ± 1.37	17.53 ± 1.62	17.63 ± 1.25	17.00 ± 1.00
	15–20	15–22	16–19	16–18
Hind foot length	14.73 ± 1.35	14.85 ± 1.97	16 ± 0.082	14.67 ± 2.31
	12–17	10–17	15–17	12–16
	***n* = 11**	***n* = 12**	***n* = 7**	***n* = 2**
Greatest length of the skull	20.54 ± 1.34	21.21 ± 0.65	21.53 ± 0.48	20.62 ± 0.08
	18.66–21.99	20.16–22.04	20.69–22.06	20.56–20.67
Braincase height	7.25 ± 0.43	7.74 ± 0.20	7.39 ± 0.59	7.98 ± 0.59
	6.80–7.86	7.42–8.01	6.61–8.23	7.56–8.39
Skull Basal length	16.59 ± 1.09	17.01 ± 0.22	17.83 ± 1.13	16.83 ± 0.11
	18.87–21.99	16.59–17.28	16.11–18.96	16.75–16.91
Palatal length	9.97 ± 0.63	10.17 ± 0.28	10.51 ± 0.39	9.72 ± 0.10
	15.06–18.25	9.87–10.68	9.89–11.1	9.65–9.79
Zygomatic breadth	10.60 ± 0.55	11.09 ± 0.31	10.84 ± 0.20	10.90 ± 0.07
	9.9–11.36	10.64–11.49	10.59–11.15	10.85–10.95
Upper toothrow length	9.92 ± 0.55	9.97 ± 0.29	10.51 ± 0.38	9.91 ± 0.13
	9.26–10.6	9.34–10.42	10.03–10.98	9.82–10
Upper molar row length	3.64 ± 0.13	3.51 ± 0.11	3.70 ± 0.13	3.43 ± 0.25
	3.45–3.87	3.33–3.71	3.49–3.86	3.25–3.6
Breadth across upper molars	4.58 ± 0.20	4.57 ± 0.29	4.72 ± 0.20	4.63 ± 0.01
	4.27–4.82	3.92–4.94	4.47–4.92	4.62–4.63
Lower toothrow length	5.88 ± 0.30	5.74 ± 0.20	6.35 ± 0.28	5.32 ± 0.30
	5.15–6.19	5.28–6.04	5.86–6.52	5.1–5.53
Lower molar row length	3.78 ± 0.18	3.72 ± 0.11	4.02 ± 0.29	3.60 ± 0.23
	3.43–4.01	3.58–4	3.68–4.45	3.43–3.76
Mandibular length	8.99 ± 0.51	8.76 ± 0.42	9.09 ± 0.30	8.70 ± 0.18
	8.25–9.7	8.09–9.62	8.75–9.5	8.57–8.83

**TABLE 5:** Morphology comparison of genus *Vernaya.*

Species	*V. fulva*	*V. foramena*	*V.* sp1	*V.* sp2
Tail	Black hairs, long and dense; the ventral part is lighter than the dorsal part	Brown‐yellow hairs, long and dense, the dorsal and abdominal uniformity	The hair on the back of the tail is yellow from the base to two‐thirds of the tail, black from one‐third of the tail, spindle‐shaped dense hair clusters formed at the tail, and yellow hair on the abdomen	Short and sparse hairs, gray
Posterior orbital process	Absent	Absent	Absent	Present
2nd upper molar	The tooth protrusions of the second and third transverse ridges are distinctive	The tooth protrusions of the second and third transverse ridges are obviously degenerated, especially the central tooth process	The tooth protrusions of the second and third transverse ridges are distinctive	The tooth protrusions of the second and third transverse ridges are distinctive
1st lower molar	The two cusps of the additional transverse ridges are separated	The two cusps of the additional transverse ridges are connected (most of specimens)	The two cusps of the additional transverse ridges are separated	The two cusps of the additional transverse ridges are separated

**TABLE 6:** Morphology of the glans penis comparison in genus *Vernaya*.

	*V. fulva*	*V. foramena*	*V.* sp1	*V.* sp2
Outer crater	Dorsal divided into two petals	Concave obviously	Concave slightly, inner crater is visibly	Circle, not concave
Lateral and distal bacula	Cartilaginous	Cartilaginous	Cartilaginous	Bone
Proximal baculum	Thin bone, the base rhombic	Robust bone, the base diamond shaped	Robust bone, the base solder‐tipped	Bone
Urethral papilla	Dorsal papilla longer and sturdier than *V*. *foramena*	Short and thin	Long and sturdy resembles *V. fulva*	Long and sturdy
Dorsal papilla	Short and bean‐liked	Short and thin	Short and bean‐liked	Very thin and relatively long

*Vernaya foramena* was the smallest of the four species (average HBL = 63 mm, average TL = 112 mm, average HBL > 67 mm, and average TL > 124 mm). The HBL of *V*. sp2 was the longest (average HBL/TL = 0.57), the relative size of the skull was the smallest (average GLS = 20.62 mm) (Table 4), and the skull of *V*. sp1 was the largest. Dental features are less variable in this genus. The tooth formula is 1.0.0.3/1.0.0.3. The length of the first upper molar was approximately equal to the sum of the second and third upper molars (Figure 6). M^1^ and M^2^ had three transverse ridges. Except for the first transverse ridge of M^2^, the remaining transverse ridges had three longitudinal tooth cups. The third transverse ridge of M^1^ and M^2^ had an additional transverse ridge, and M^3^ had two plate transverses (Figure 6). Among them, the tooth characteristics of *V. foramena* alone showed some variations compared to those of other species: (1) The tooth cups of the second and third transverse ridges of *V. foramena* were notably degenerated and tended to be plate‐like transverse ridges (Figure 6a2), whereas the others were distinctive, with the central tooth process being the largest, followed by the lateral dentition (Figure 6b2,c2,d3). (2) The difference between the two mandibular teeth was that the two accessory processes of *V. foramena* were connected (Figure 5a7) [except for one specimen (SAF201560)], whereas the accessory processes of the other were separated (Figure 5b7,c7,d7). The dental characteristics of *V*. sp1 and *V*. sp2 were consistent with those of *V. fulva*; no substantial variations were observed.

The penises of the four species differ significantly. The dorsal area of the outer crater of *V. foramena* was concave (Figure 7a). The lateral and distal bacula were cartilaginous, the proximal baculum was a robust bone, and the base was diamond shaped. The urethral (Figure 7a2) and dorsal papillae (Figure 7a3) were short and thin, respectively. The outer carter of *V. fulva* was divided into two petals (Figure 7b); the lateral and distal bacula were cartilaginous; the proximal baculum was thin bone and the base rhombic; the dorsal papilla (Figure 7b3) was longer and sturdier than *V. foramena;* and the urethral papilla (Figure 7b2) was extremely short and bean‐like. The dorsal outer crater of *V*. sp1 was slightly concave, the inner crater was visible, the lateral and distal bacula were cartilaginous, the proximal baculum was a robust bone, and the base was solder‐tipped. The dorsal papilla (Figure 7c3) was long and sturdy, resembling *V. fulva*, and the urethral papilla (Figure 7c2) was extremely short and bean‐like. The dorsal outer crater of *V*. sp2 was circular, not concave; the lateral, distal, and proximal bacula were bone; however, the lateral bacula was only slightly ossified, and the distal and base parts of the proximal baculum were semi‐circular. The dorsal papilla (Figure 7d3) was long and sturdy, and the urethral papilla (Figure 7d2) was very thin and relatively long.

## 4 DISCUSSION

In 1980, Wang et al. (1980) proposed that *V. foramena* was a new species of the genus *Vernaya* (Wang et al., 1980). He suggested that the distinguishing features between *V. foramena* and *V. fulva* were mainly based on two points: (1) The frontal foramen ovale of *V. foramena* were clearly exposed and arranged in parallel, whereas the foramen ovale of *V. fulva* were hidden under a thin film and arranged in an “eight” shape. (2) The two cusps of the additional transverse ridge of the posterior margin of the mandibular M_1_ of *V. foramena* were connected, whereas those of *V. fulva* were separated. Later, Li and Wang (1995) compared specimens from Zhouqu County in Gansu and Fengxian in Shaanxi and found that the two discriminatory characteristics mentioned by Wang were unstable in specimens from different locations. They concluded that, in addition to the visible difference in hair color between *V. foramena* and *V. fulva*, the presence of the frontal foramen ovale and whether the two tooth tips of the additional transverse ridge of the M_1_ posterior edge were connected vary from individual to individual.

According to our results, nearly half of the frontal foramen ovale in *V. foramena* was not exposed, whereas two in *V. fulva* were exposed. Therefore, we agree with Li and Wang (1995) that exposure to the foramen ovale differs between individuals. In addition, the connection or separation of the two dentary tips of the additional transverse ridges of M_1_ distinguishes *V. fulva* from *V. foramena*.

The evolution of animals was closely related to the climate. Research has shown that the diversity of rodents exhibited notable alterations with ancient climate change (Li et al., 2022). The apparent alteration of Pleistocene climates may have contributed to the expansion and divergence of the species (Song et al., 2013; Tagliacozzo et al., 2016; Xing et al., 2013). According to our analysis, the origin of species within the genus *Vernaya* can be traced back to the middle Pliocene, with species differentiation mainly occurring during the Pleistocene (*V. foramena* diverged from *V. fulva* and *V*. sp1 2.49 Ma, 95% CI = 0.8–6.23, and *V. fulva* and *V*. sp1 diverged 1.57 Ma, 95% CI = 0.47–3.99). We speculate that this was related to climate change during the Pleistocene. The species formation and evolution of the genus *Vernaya* were closely related to the uplift of the Qinghai–Tibetan Plateau (QTP) and its biological characteristics. The QTP uplifted several times; the most intense one was during the Qinghai–Tibet movement (uplifting from 3.4 Ma, strong uplifting from 2.5 to 1.7 Ma) and the Kun–Huang Movement (1.2–0.6 Ma) (An et al., 2001; Shi et al., 1999; Zheng et al., 2000). This movement has changed the surrounding climatic patterns and topographic structures, providing strong support for the formation, distribution, and dispersal of species, and has shaped modern biodiversity. The earliest divergence of the genus can be traced back to 3.36 million years, and the subsequent divergence of several species occurred in 2.49 Ma (*V. foramena*) and 1.57 Ma (*V. fulva* and *V*. sp1), respectively. These temporal events almost coincided with the uplift periods of the Tibetan Plateau. Thus, we hypothesized that the formation of these four species was caused by the overall uplift of the QTP, resulting in geographic isolation and the prevention of genetic exchange. Its small individual size, arboreal living habits, and weak diffusion ability further hinder gene exchange and the formation of new species.

## 5 TAXONOMIC ACCOUNT

Historically, the genus *Vernaya* has been considered monotypic. In this study, we conducted a comprehensive reassessment of species diversity within the *Vernaya* genus by integrating molecular and morphological data. The results revealed a significant underestimation of the species diversity in the genus prior to this study. Based on these results, a thorough taxonomic revision of *Vernaya* species was performed. In summary, four species are currently recognized within the genus, the first being *V. fulva*, which was collected from areas east of the Nujiang River in Yunnan Province. The second most common species was *V. foramena*, which was previously considered a subspecies of *V. fulva*. However, our collection of specimens from the type locality of *V. foramena* revealed characteristics that differentiate it from *V. fulva*. Therefore, *V. foramena* should be recognized as a separate, valid species. In terms of morphological and molecular differences, individuals collected from the Meigu and Gongga Mountains in Sichuan were identified as a new species, designated *V. meiguites*. Additionally, specimens obtained from regions west of the Nujiang River in Yunnan were categorized as a novel species, referred to as *V. nushanensis*. This taxonomic update contributes to a better understanding of the diversity and evolutionary history of the *Vernaya*.

**Family Muridae Gray, 1821.**

**Genus *Vernaya* Anthony, 1941.**

***Vernaya fulva Anthony, 1941*.**

*Chiropodomys fulvus* Allen, 1927:11.

*Vandeleuria dumeticola* Allen, 1940:1048.

*Vernaya fulva* Anthony, 1941:110.

**Holotype:** Specimen number: 43989, an adult female collected by R. C. Andrews and E. Heller in 1916. The specimen was deposited at the American Museum of Natural History.

**Type locality:** Yinpankai, Mekong River, western Yunnan, China, 9000 feet altitude.

**Common names:** Climbing mouse, 攀鼠 (Panshu).

**Diagnosis:** HBL averages approximately half of the TL (slightly shorter than or slightly longer than half of the TL). Body light brownish yellow, entire dorsal tail covered with black hairs, ventral part notably lighter than dorsal part to distinguish it from congeners (Figure 5b4).

**Remarks:** A medium‐sized species of *Vernaya*. Allen (1927) collected a specimen named *Chiropodomys fulvus*, which was later considered synonymous with *Vandeleuria dumeticola* (Allen, 1940). Anthony (1941) changed the name of the species to *Vernaya fulva*. At present, this species is found in Yunnan Province alone and forms two groups with the neighboring *V. nushanensis* sp. nov. We speculate that communication is blocked by rivers, which leads to population differentiation. Geographical distribution must be determined by expanding the number of sampling points.

**Distribution:** *Vernaya fulva* is distributed in western Yunnan (Allen, 1927), China, west of the Lancang River (upper Mekong River in China), and extends into northern Myanmar (Anthony, 1941). The specimens were collected between 2100 and 3300 m.

***Vernaya foramena WANG, 1980*.**

*Vernaya foramena* WANG, 1980:26.

*Vernaya fulva foramena* Li, 1995:325–328.

**Holotype:** Specimen number: 75003, adult female collected in 1975. The specimen was deposited at China West Normal University.

**Type locality:** Wanglang, Pingwu, Sichuan Province, China (2490 m a.s.l.).

**Common names:** Apparent climbing mouse, 显孔攀鼠 (Xiankong Panshu).

**Diagnosis:** *Vernaya foramena* is the smallest species in the genus *Vernaya*. The body is brown, the abdomen is white, and the hair base is gray. It is distinguished from other species based on the following features: (1) the tail is covered with long and dense brownish‐yellow hairs; (2) the cusps of the second and third transverse ridges of the second upper molars are visibly reduced, especially the central cusp; and (3) the two cusps of the additional transverse ridges of the first lower molars are connected (Figure 5a1–a4 and 6a1–a8).

**Description:** *Vernaya foramena* is the smallest species in the genus *Vernaya*, with a head‐body length of 55–73 mm (average 63 mm), tail length of 100–125 mm (average 112 mm) (HBL/TL = 0.56), average hind foot length of 15 mm, and average ear length of 17 mm. Dorsal hair is long and soft; the hair base is gray; the hair tip is brown; the ventral hair base is light gray; the hair tip is white and partly dyed yellowish; the throat pure is white; and the dorsal and ventral color boundaries are notable. Dorsal and ventral tails are brownish yellow; the ventral side is slightly lighter than the dorsal side; the tail end is covered with brownish yellow long hair; and the tail end has a few terminal hairs (Figure 5a1–a4).

The cranial part of the brain is round, the cranial vault is slightly convex, the anterior margin of the zygomatic plate is straight, and the zygomatic arch is curved inward. The nasal bone is flushed with the anterior border of the zygomatic arch, and the foramen ovale is visible or faint. The auditory vesicles are narrow, long, and convex (Figure 6a1,a3).

The maxillary incisors are orange and curved. The first upper molar is the largest, with complete three transverse ridges and three longitudinal cusps; the second molar has degenerated longitudinal cusps of the three transverse ridges, especially the middle cusps (t^2^, t^5^, and t^8^); the first transverse ridge tends to be straight; and both the first and second upper molars have additional transverse ridges on the posterior margin (Figure 6a2). The first lower molar is the largest, with three transverse ridges and three longitudinal cusps; the third transverse ridge has additional transverse ridges, and the two cusps of the additional transverse ridges are connected in most specimens (one specimen is separated); the second molars have three longitudinal cusps of the first and second transverse ridges, and the cusps of the third transverse ridge are degenerated. M_3_ possesses two transverse ridges and degenerate cusps (Figure 6a7).

The glans penis (Figure 7a1) is pole‐like and slender. The dorsal outer crater is visibly concave (Figure 7a). The urethral lappet has three forks, with the middle being the tallest (Figure 7a2). The lateral and distal bacula are cartilaginous, the proximal baculum is a robust bone, and the base is diamond shaped. The urethral (Figure 7a2) and dorsal papillae (Figure 7a3) are short and thin, respectively.

**Comparisons:** *Vernaya foramena* was found to be the smallest species in the genus *Vernaya* (average HBL, 63 mm; average TL, 112 mm). The tail is covered with long, dense brownish yellow hair, dorso‐ventrally uniform (Figure 5a4); the tail of *V. fulva* is black (Figure 5b4); the abdomen is lighter in color than the dorsum; the tail of *V. meiguites* sp. nov. becomes black from two‐thirds of the way up, with black tufts of hair at the tip (Figure 5c4); and *V. nushanensis* sp. nov. has sparse and short gray hairs (Figure 5d4). It differs from other species by the two tips of the additional transverse ridge of the M_1_ of the lower jaw being connected (Figure 6a7). In *V. nushanensis* sp. nov., the orbital rim forms a relatively smooth arc (Figure 6a4,d4).

**Comments:** This species was found to be the smallest in size in the genus *Vernaya*, with an HBL average of 63 mm and aTL of 112 mm. In 1980, Wang collected a group of specimens from Wanglang, Sichuan, and identified them as *V. foramena*; later, Li and Wang (1995) denied the status of independent valid species and proposed *V. foramena* as a geographic subspecies of *V. fulva*. We compared specimens from Wanglang and surrounding areas in Sichuan and proposed that the species status of *V. foramena* be restored.

**Distribution:** *Vernaya foramena* is distributed in the Qinling Mountains of northern Sichuan (Wang et al., 1980), southern Gansu, and southwestern Shaanxi (Li & Wang, 1995). It has also been found in central Sichuan, the Qionglai Mountain District in western Sichuan, and northeastern Chongqing. The altitude range was 1200–3000 m.

***Vernaya meiguites*** Zhao SP, Li BB, Wang XM, Jiang XL, Liu SY, Chen SD, sp. *Nov*.

**Holotype:** Specimen number SAF201652 (field number: 2020MG045), an adult male collected by Binbin Li and Miao Hu in 2020. Specimens was deposited at the Sichuan Academy of Forestry.

**Type locality:** Hongxi, Meigu, Sichuan Province, China (103.051° E, 28.64858 N, 2616 m a.s.l.).

**Paratypes:** An adult female (SAF201653) collected with the holotype in Hongxi, Meigu, Sichuan, China (103.051E, 28.64858 N, 2616 m a.s.l.) and deposited at the Sichuan Academy of Forestry; eight specimens of unknown sex (MCU104071, MCU104072, MCU104073, MCU104074, MCU104075, MCU104076, MCU104077, and MCU104078) collected from Hongxi, Meigu, Sichuan, China and deposited at the Sichuan University. An adult male (SAF220266) was collected from the Gongga Mountains, Sichuan, China, in 2022 (101.523272° E, 29.124803 N, 3300 m a.s.l.) and deposited at the Sichuan Academy of Forestry.

**Common names:** Meigu climbing mouse, 美姑攀鼠 (Meigu Panshu).

**Diagnosis:** Slender body, the HBL averages approximately half of the TL (slightly shorter than or slightly longer than half of the TL), the smallest proportion in *Vernaya* but the largest in the skull and relatively flat in this genus. Following are the physical features of this species: brownish yellow body, relatively long ears (average 16 mm), tail tip tufted with long black hair, spindle shaped (Figure 5c1–c4). The upper and lower mandibular teeth are longer in this species.

**Description:** Slim figure, head‐body length of 58–76 mm (average 67 mm), tail length of 125–132 mm (average 128 mm) (HBL/TL = 0.52), average hind foot reaches 18 mm, and average ear length is 16 mm. Back hair long and soft, reddish brown, belly hair tip yellowish, hair base black–gray (Figure 5c1–c3). The dorsal part of the tail is light black, darkening gradually from half of the tail; the ventral part is brownish yellow, covered with brown hair; the dorsal hair is slightly longer; black hair appears from half of the tail; and the tail tip is tufted with long black hairs (Figure 5c4). The tail is approximately twice as long as the HBL, the longest tail length among *Vernaya*. The hind feet are long, and the dorsal feet are brown.

The skull is the longest in this genus (21.53 ± 0.48 mm), and the braincase top is slightly convex. The front edge of the zygomatic plate was straight, and the middle part of the zygomatic arch was bent inward. Nasal bones slightly exceed the line of the anterior margin of the zygomatic arch. Both sides of the orbital rim form relatively smooth arcs. (Figure 6c3). The auditory bulla is large and bulging (Figure 6c1).

The maxillary incisors are orange and curved. There are three molars in the maxilla: M^1^ is the largest, followed by M^2^_,_ and then M^3^. M^1^ and M^2^ possess three transverse ridges, which form three longitudinal tooth cups, except for the first transverse ridge of M^2^. The tooth cups are pronounced and “W” shaped (Figure 6c2). The first transverse ridge of M^2^_,_ without the central odontoid process and the inner and outer odontoid processes, becomes uniformly large outward from the middle (in one specimen, the tips were separated). M^3^ was the smallest, with two transverse plates (Figure 6c2). The M_1_ of mandibular is the largest, with three transverse ridges and nine odontoid processes, with a triangular lobule on the anterior margin of the first transverse ridge and an additional transverse ridge on the third transverse ridge, the two tips of this additional transverse ridge are separated (Figure 6c7); M_2_ has two transverse ridges with six odontoid processes and a plate‐like third transverse ridge; and the lateral denticles (t_1_ and t_4_) of the first and second molars are shifted downward. The third molars are the smallest, with two plate‐like transverse ridges (Figure 6c7).

The glans penis (Figure 7c1) is pole‐like and slender. The dorsal side of the outer crater is slightly concave, and the inner crater is visible (Figure 7c1). The urethral lappet has three forks, with the middle being the tallest (Figure 7c2). The dorsal papilla (Figure 7c3) is extremely short and bean‐like; the lateral and distal bacula are cartilaginous; the proximal baculum is a robust bone; and the base is solder‐tipped (Figure 7c4).

**Comparisons:** Compared to other species in this genus, the relative TL was the longest (average HBL/TL = 0.52). The average HFL was greater than 17 mm, and the EL was 16 mm. The tail is back, the belly is brown, half of the tail has black hair, and the tail tip is tufted with long black hairs (Figure 5c4). It differs from *V. foramena* by the two tips of the additional transverse ridge of M_1_ of the lower jaw being separated (Figure 6a7,c7). In *V. nushanensis* sp. nov., the orbital rim forms a relatively smooth arc (Figure 6c5,d5).

**Etymology:** The new species is named after its type locality, Meigu County, where the Meigu Dafengding National Nature Reserve is located. This area is in the transitional zone between the Sichuan Basin and the Yunnan–Guizhou Plateau, the southeastern edge of the Qinghai–Tibet Plateau, and the middle of the Hengduan Mountains. The mountainous area of southwestern China is one of the key areas of global biodiversity and one of the most complete areas of subtropical mountain flora and fauna resources in southwestern China. Naming new species at the county level reflects the high value of biodiversity conservation. Thus, we suggest “Meigu climbing mouse” as the English common name and “美姑攀鼠 (Meigu Panshu)” as the Chinese common name.

**Distribution:** Distributed in Meigu Dafengding National Nature Reserve, Mabian Dafengding National Nature Reserve, and adjacent areas. New species also occur in the Gongga Mountains, which are the junction of the Kangding, Luding, Jiulong, and Shimian counties in the middle of the Hengduan Mountains.

***Vernaya nushanensis*** Zhao SP, Liu YX, Jiang XL, Liu SY, Chen SD, sp. *nov*.

**Holotype:** Specimen number SAF19287, an adult male collected on September 9, 2019, by Rui Liao. Specimens were deposited at the Sichuan Academy of Forestry.

**Type locality:** Caojian, Dali, Yunnan Province, China (99.11484° E, 25.75724 N, 2500 m a.s.l.).

**Paratypes:** Two adult males (KIZ XMS170115 and XMS170200) were collected from Xuemeng Mountain in April 2017 (98.93872° E, 25.96119 N, 3470 m a.s.l.). All specimens were deposited at the Kunming Institute of Zoology; the skull of KIZ XMS170115 was missing.

**Common names:** Nushan climbing mouse, 怒山攀鼠 (Nushan Panshu).

**Diagnosis:** HBL averages approximately two‐thirds as long as TL (slightly shorter than or slightly longer than two‐thirds of TL), but the skull is relatively smallest. Following are the physical features of this species: brown body, grayish white tail, covered with sparse white short hair (Figure 54d). The orbit with the posterior orbital process was distinct from other species of the *Vernaya* (Figure 6d5).

**Description:** Slim figure, head‐body length of 69–75 mm (average 71 mm), tail length of 113–133 mm (average 125 mm) (HBL/TL = 0.57), average hind foot reaches 17 mm, and average ear length is 15 mm. The ears are bare with fluffy attachments. The dorsum of the foot is brownish yellow, and the tips of the toes have white hair. The top of the head is light brown–gray from the end of the muzzle to the posterior edge of the eyes. The tips of the hairs on the back of the body are light brownish gray, the ventral hair is white, the tip locally light yellow, the hair base is grayish black, and the throat hair is white (Figure 5d1–d3). The tail is long and slender, grayish white, consistent with the upper and lower, pencil shaped, covered with sparse white short hair, with a few longer light blackish hairs at the tip (Figure 5d4).

The skull is slender (20.62 ± 0.08 mm) and small relative to HBL in this genus. The braincase is relatively high and rounded, approximately spherical in top view (Figure 6d3). The zygomatic arch is weak and wide. Nasal bones are long, exceeding the line of the anterior margin of the zygomatic arch (Figure 6d3). The supraorbital ridge expands laterally at the posterior margin of the orbit, forming a curved bony plate (Figure 6d3,d5). The joint suture in the middle of the frontal bone is straight, with sharp angles forming on both sides (Figure 6d3). The palatal foramen is short, the posterior margin does not reach the line of the anterior margin of the molars, and the auditory vesicles are large and round (Figure 6d1).

The teeth of the entire taxon are 1.0.0.3/1.0.0.3. The maxillary incisors are orange and curved. There were three molars in the maxilla, and the length of the first upper molar is approximately equal to the sum of the second and third upper molars. M^1^ and M^2^ possess three transverse ridges, which have three longitudinal tooth cups, except for the first transverse ridge of the M^2^. The middle process is the largest, and the external tooth protrusion of the third transverse ridge is upward. The first transverse ridge of M^2^ without the central odontoid process and inner and outer odontoid processes downward. The third transverse ridge of M^1^ and M^2^ is an additional transverse ridge that emerges from the junction of the medial and middle tooth protrusions and follows the outer dentary process upward. M^3^ is the smallest, with two transverse plates (Figure 6d3). The mandibular first molar is the largest, with three transverse ridges and three longitudinal rows of denticles, a triangular lobule on the anterior margin of the first transverse ridge, and an additional transverse ridge on the third transverse ridge; the two tips of this additional transverse ridge are separated; the second molar has three transverse ridges, three longitudinal rows of denticles, and a plate‐like third transverse ridge; and the third molars are the smallest, with two plate‐like transverse ridges (Figure 6d9).

The glans penis (Figure 7d1) is pole‐like and slender. The dorsal side of the outer crater is circular and nonconcave (Figure 7d1). The urethral lappet has three forks; the middle is the highest and smoothest columnar, and the forks on the sides are short and small (Figure 7d2). The dorsal papilla (Figure 7d3) is notable and tall; the lateral, distal, and proximal bacula are bone, but the lateral bacula is slightly ossified, and the distal and base parts of the proximal baculum are semi‐circular (Figure 7d4).

**Comparisons:** The shape and skull ratio were similar to *V. foramena*; however, the full length was much larger, and the two tips of the additional transverse ridge of the third transverse ridge of the mandibular first molar were separated (Figure 6d9). The HBL was the longest in the genus, but the skull was relatively small, with a relatively high BH. It differs from other species in that it has a lighter color on the dorsal body, a uniform gray‐white tail, short and sparse hair (Figure 5d4), and a posterior orbital process (Figure 6d5).

**Etymology:** The new species was found in the Nushan Mountain range, which is bounded by the Nu and Lancang Rivers and is rich in animal and plant resources. We suggest “Nushan climbing mouse” as the English common name and “怒山攀鼠 (Nushan Panshu)” as the Chinese common name.

**Distribution:** Currently found in Xuemeng Mountain, Lushui, Caojian, Dali, Yunnan, China. The Lancang River (the upper Mekong River in China) is likely the eastern boundary of its distribution. This species inhabits a wide range of elevations (2700–3750 m), and the type locality is an azalea shrub habitat with an elevation >3000 m.

### 5.1 Key to genus *Vernaya*

1. The tooth cups of the second and third transverse ridges are clearly degenerated and tend to be plate‐like transverse ridges, and the two accessory processes of the mandible are connected*Vernaya foramena*

The tooth cups of the second and third transverse ridges are distinct, with the central tooth process being the largest, followed by the lateral dentition; the two accessory processes of the mandibular are separated2

2. Tail covered with short, sparse hair, gray; supraorbital crest expanded to the sides at the posterior margin of the orbit, forming an arched bony plate*Vernaya meiguites*

Tails covered with dense long hair, yellow or black; supraorbital crest arcing inward3

3. Tail tip tufted with long black hair; coronoid process low and bluntly rounded…………………………………………………*Vernaya nushanensis*.

The tip of the tail is slightly longer than hair toward the base but does not form a tuft; the coronoid process long and is pointed*Vernaya fulva*.

## 6 CONCLUSIONS

In this study, we investigated the phylogenetic relationships and evolutionary history of *Vernaya* using molecular and morphological data. The molecular analysis and morphological results supported the division of the genus into four valid independent species: *V. fulva*, *V. foramena*, *V. meiguites*, and *V. nushanensis*. In addition, our divergence time tree suggests that the divergence of the species of the genus *Vernaya* may be related to Pleistocene climate change and the uplift of the Tibetan Plateau. Finally, we comprehensively reviewed the species in the genus *Vernaya* and produced a key table.

## AUTHOR CONTRIBUTIONS

**Songping Zhao:** Formal analysis (lead); investigation (lead); methodology (lead); software (lead); writing – original draft (lead); writing – review and editing (equal). **Xuming Wang:** Data curation (equal); formal analysis (equal); investigation (equal); methodology (equal); writing – review and editing (equal). **Binbin V. Li:** Formal analysis (equal); investigation (equal); methodology (equal); writing – review and editing (equal). **Liang Dou:** Data curation (equal); investigation (equal); resources (equal). **Yingxun Liu:** Data curation (equal); investigation (equal); resources (equal). **Siyu Yang:** Formal analysis (equal); investigation (equal). **Ronghui Fan:** Formal analysis (equal); investigation (equal); software (equal). **Quan Li:** Data curation (equal); investigation (equal); resources (equal). **Yong Jiang:** investigation (equal); resources (equal). **Rui Liao:** Data curation (equal); investigation (equal); resources (equal). **Miao Hu:** Investigation (equal); resources (equal). **Xuelong Jiang:** Formal analysis (equal); funding acquisition (lead); investigation (equal); methodology (equal); resources (lead); supervision (equal); writing – review and editing (equal). **Shaoying Liu:** Data curation (lead); formal analysis (equal); funding acquisition (lead); investigation (equal); methodology (equal); resources (lead); software (equal); supervision (lead); writing – review and editing (lead). **Shunde Chen:** Formal analysis (equal); funding acquisition (lead); investigation (equal); methodology (equal); resources (lead); supervision (lead); writing – review and editing (lead).

## CONFLICT OF INTEREST STATEMENT

The authors declare no conflict of interest.

## Supporting information


Figure S1.

Table S1.

Table S2.

Table S3.

Table S4.

Table S5.

## Data Availability

Data from the results of this study are freely available in GenBank at the NCBI website (https://www.ncbi.nlm.nih.gov) (Accession numbers OR282742–OR282761, OR333541–OR333696).
