# Supplementary figures and images for: Molecular phylogeny and taxonomy of the genus Vernaya (Mammalia: Rodentia: Muridae) with the description of two new species
Source: Ecol Evol. 2023 Nov 9;13(11):e10628. doi: 10.1002/ece3.10628 (PMC10636494; doi:10.1002/ece3.10628)

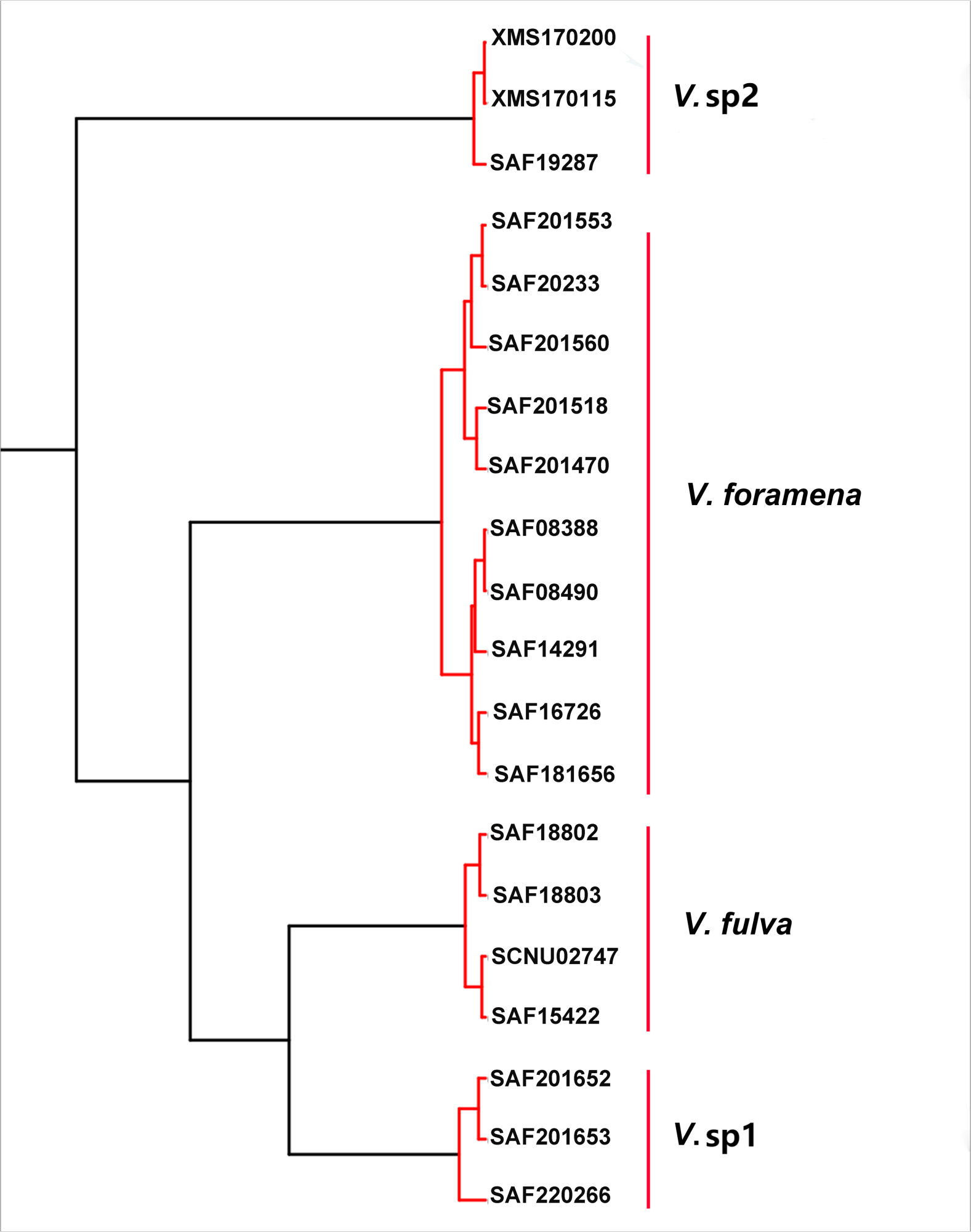

Supplement: Supplementary file 1 — Figure S1. [file ECE3-13-e10628-s005.png]
