# Supplementary material for: Molecular phylogeny and taxonomy of the genus Vernaya (Mammalia: Rodentia: Muridae) with the description of two new species
Source: Ecol Evol. 2023 Nov 9;13(11):e10628. doi: 10.1002/ece3.10628 (PMC10636494; doi:10.1002/ece3.10628)
Supplement: Supplementary file 4 — Table S3. [file ECE3-13-e10628-s003.docx]

Supplementary table 3. The measurement data on the appearance and skull morphology of the specimens of the genus *Vernaya* in this study

| Species | Museum number | Sex | W | HBL | TL | HFL | EL | SGL | BH | SBL | PL | ZB | UTL | LUM | BUM | LTL | LLM | ML |
| --- | --- | --- | --- | --- | --- | --- | --- | --- | --- | --- | --- | --- | --- | --- | --- | --- | --- | --- |
| *V. foramena* | SAF181656 | - | 14 | 73 | 122 | 17.5 | 15 | 21.82 | 7.64 | 17.73 | 10.51 | 11.36 | 10.6 | 3.87 | 4.82 | 6.19 | 4 | 9.7 |
|  | SAF14291 | female | 8 | 60 | 100 | 15 | 13 | - | - | - | - | - | - | - | - | - | - | - |
|  | SAF16726 | female | 8 | 60 | 103 | 15 | 15 | 18.66 | 6.8 | 15.06 | 9.18 | 10.21 | 9.18 | 3.45 | 4.27 | 5.15 | 3.43 | 8.4 |
|  | SAF08388 | female | 10 | 67 | 117 | 16 | 16 | 20.54 | 7.66 | 16.21 | 10.13 | 10.61 | 9.61 | 3.48 | 4.45 | 5.67 | 3.63 | 8.87 |
|  | SAF08490 | male | 12 | 67 | 125 | 17 | 17 | - | - | - | - | - | 10.13 | 3.58 | 4.73 | 5.88 | 4.01 | 9.01 |
|  | SAF201560 | female | - | 55 | 106 | 17 | 15 | 19.54 | 7.28 | 15.54 | 9.32 | 10.07 | 9.64 | 3.67 | 4.53 | 5.86 | 3.93 | 8.66 |
|  | SAF201553 | male | - | 60 | 113 | 17 | 15 | 21.37 | 6.98 | 16.99 | 10.16 | 10.5 | 10.12 | 3.63 | 4.55 | 6.05 | 3.73 | 8.99 |
|  | SAF201518 | - | - | 54 | 105 | 16 | 15 | 18.87 | 6.87 | 15.95 | 9.33 | 9.9 | 9.26 | 3.6 | 4.36 | 5.87 | 3.85 | 8.25 |
|  | SAF201470 | male | - | 65 | - | 17 | 15 | 21.49 | 7.44 | 16.95 | 10.2 | 10.82 | 9.96 | 3.66 | 4.65 | 6.02 | 3.72 | 9.47 |
|  | SAF20233 | - | - | 66 | 116 | 17 | 12 | 21.99 | 7.86 | 18.25 | 10.91 | 11.34 | 10.76 | 3.79 | 4.82 | 5.98 | 3.69 | 9.73 |
| *V. fulva* | SAF15422 | male | 10.4 | 65 | 120 | 17 | 17 | 21.04 | 8.01 | 16.59 | 9.87 | 10.83 | 9.65 | 3.59 | 4.3 | 5.93 | 3.62 | 8.59 |
|  | SAF18802 | female | 10 | 59 | 131 | 18 | 15 | - | - | - | - | - | 9.92 | 3.44 | 3.92 | 5.61 | 3.58 | 8.67 |
|  | SAF18803 | male | 11 | 71 | 143 | 19 | 16 | 21.42 | 7.88 | 16.97 | 10.23 | 10.64 | 10.02 | 3.71 | 4.72 | 6.04 | 3.83 | 9.62 |
|  | SCNU02747 | - | 10.04 | 62 | 112 | 17 | 16 | 20.31 | 7.42 | 16.93 | 9.98 | 10.71 | 9.93 | 3.58 | 4.61 | 5.66 | 4 | 9.11 |
|  | 018995 | male | 11 | 63 | 112 | 15 | 14 | 20.16 | 7.86 | 16.84 | 9.90 | 11.04 | 9.86 | 3.33 | 4.64 | 5.28 | 3.63 | 8.16 |
|  | 014782 | male | 12 | 72 | 126 | 17 | 16 | 21.45 | 7.62 | 16.97 | 9.96 | 11.27 | 9.99 | 3.44 | 4.56 | 5.69 | 3.67 | 8.81 |
|  | 014781 | female | 11 | 66 | 121 | 22 | 15 | 21.89 | 7.66 | 17.15 | 10.39 | 11.12 | 10.32 | 3.53 | 4.94 | 5.86 | 3.77 | 8.62 |
|  | 014778 | female | 10.5 | 70 | 126 | 18 | 17 | 21.56 | 7.98 | 17.04 | 10.48 | 11.20 | 10.07 | 3.51 | 4.83 | 5.83 | 3.73 | 8.83 |
|  | 014787 | - | 7 | 55 | 116 | 18 | 15 | - | - | - | - | - | 9.34 | 3.39 | - | 5.61 | 3.68 | 8.09 |
|  | 014788 | female | 18 | 77 | 142 | 19 | 6 | - | - | - | 10.68 | - | 10.42 | 3.47 | 4.32 | 5.71 | 3.65 | - |
|  | 007619 | female | 17 | 72 | 130 | 16 | 15 | 21.04 | 7.57 | 17.3 | 9.98 | 11.47 | 9.86 | 3.47 | 4.71 | 5.7 | 3.72 | 8.93 |
|  | 005009 | - | - | 80 | 132 | 17 | 10 | 22.04 | 7.68 | 17.28 | 10.23 | 11.49 | 10.26 | 3.62 | 4.75 | 5.92 | 3.73 | 8.94 |
|  | 014780 | female | 13.5 | 74 | 122 | 17 | 13 | - | - | - | - | - | - | - | - | - | - | - |
|  | 014784 | male | 12.7 | 75 | 130 | 16 | 15 | - | - | - | - | - | - | - | - | - | - | - |
|  | 014785 | female | 9.5 | 54 | 115 | 16 | 11 | - | - | - | - | - | - | - | - | - | - | - |
|  | 014786 | male | 11.2 | 68 | 130 | 19 | 14 | - | - | - | - | - | - | - | - | - | - | - |
|  | 014779 | male | 11 | 68 | 115 | 17 | 17 | - | - | - | - | - | - | - | - | - | - | - |
|  | 014783 | female | - | - | - | - | - | - | - | - | - | - | - | - | - | - | - | - |
|  | 015600 | - | - | - | - | - | - | - | - | - | - | - | - | - | - | - | - | - |
| *V. meiguites* | SAF201652 | female | - | 65 | 128 | 18 | 17 | 21.69 | 6.82 | 17.16 | 10.42 | 10.76 | 10.27 | 3.73 | 4.61 | 6.23 | 3.92 | 9.11 |
|  | SAF201653 | female | - | 58 | 125 | 19 | 16 | 20.69 | 7.41 | 16.11 | 9.89 | 10.61 | 10.11 | 3.61 | 4.47 | 6.18 | 4.01 | 9.5 |
|  | SAF220266 | male | 13.6 | 76 | 132 | 17.5 | 15 | 21.31 | 6.61 | 17.41 | 10.36 | 11.15 | 10.03 | 3.49 | 4.47 | 5.86 | 3.68 | 8.75 |
|  | MSCU104071 | - | - | - | - | - | - | - | - | - | 10.91 | 10.59 | 10.86 | 3.8 | 4.84 | 6.48 | 3.68 | 8.81 |
|  | MSCU104072 | - | - | - | - | - | - | - | - | - | - | - | - | - | - | - | - | - |
|  | MSCU104073 | - | - | - | - | - | - | - | - | - | - | - | - | - | - | - | - | - |
|  | MSCU104074 | - | - | - | - | - | - | 21.86 | 8.23 | 18.87 | 10.44 | 10.93 | 10.72 | 3.77 | 4.85 | 6.52 | 4.1 | 9.11 |
|  | MSCU104075 | - | - | 69 | 126 | 16 | 16 | 21.59 | 7.61 | 18.46 | 10.44 | 10.99 | 10.61 | 3.67 | 4.88 | 6.45 | 4.45 | 9.02 |
|  | MSCU104076 | - | - | - | 140 | - | - | 22.06 | 7.65 | 18.96 | 11.1 | 10.86 | 10.98 | 3.86 | 4.92 | 6.71 | 4.27 | 9.36 |
|  | MSCU104077 | - | - | - | - | - | - | - | - | - | - | - | - | - | - | - | - | - |
|  | MSCU104078 | - | - | - | - | - | - | - | - | - | - | - | - | - | - | - | - | - |
| *V. nushanensis* | SAF19287 | male | 13 | 70 | 113 | 16 | 12 | 20.67 | 8.39 | 16.75 | 9.65 | 10.85 | 10 | 3.6 | 4.62 | 5.1 | 3.76 | 8.57 |
|  | XMS170200 | male | 9.5 | 69 | 128 | 18 | 16 | 20.56 | 7.56 | 16.91 | 9.79 | 10.95 | 9.82 | 3.25 | 4.63 | 5.53 | 3.43 | 8.83 |
|  | XMS170115 | male | 12.3 | 75 | 133 | 17 | 16 | - | - | - | - | - | - | - | - | - | - | - |
