# Supplementary material for: Molecular phylogeny and taxonomy of the genus Vernaya (Mammalia: Rodentia: Muridae) with the description of two new species
Source: Ecol Evol. 2023 Nov 9;13(11):e10628. doi: 10.1002/ece3.10628 (PMC10636494; doi:10.1002/ece3.10628)
Supplement: Supplementary file 5 — Table S4. [file ECE3-13-e10628-s002.docx]

Supplementary table 4. Posterior probabilities for the 4 putative species using different priors for model parameters

| (a) ~ G (2, 2000) and τ ~ G (2, 2000) | | | | | | |
| --- | --- | --- | --- | --- | --- | --- |
| nDNA-algorithm0 | speciesdelimitation=1 0 2 | speciesdelimitation=1 0 10 | speciesdelimitation=1 0 20 | speciesdelimitation=1 0 2 | speciesdelimitation=1 0 10 | speciesdelimitation=1 0 20 |
|  | heredity= 1 4 4 | heredity= 1 4 4 | heredity= 1 4 4 | locusrate = 1 10.0 | locusrate = 1 10.0 | locusrate = 1 10.0 |
|  | P[ 4] = 1.00000 | P[ 4] = 1.00000 | P[ 2] = 0.00220 | P[ 4] = 1.00000 | P[ 4] = 1.00000 | P[ 4] = 1.00000 |
|  |  |  | P[ 3] = 0.00390 |  |  |  |
|  |  |  | P[ 4] = 0.99390 |  |  |  |
| nDNA-algorithm1 | speciesdelimitation=1 1 1 0.5 | speciesdelimitation=1 1 1.5 1 | speciesdelimitation=1 1 2 2 | speciesdelimitation=1 1 1 0.5 | speciesdelimitation=1 1 1.5 1 | speciesdelimitation=1 1 2 2 |
|  | heredity= 1 4 4 | heredity= 1 4 4 | heredity= 1 4 4 | locusrate = 1 10.0 | locusrate = 1 10.0 | locusrate = 1 10.0 |
|  | P[ 4] = 1.00000 | P[ 4] = 1.00000 | P[ 4] = 1.00000 | P[ 4] = 1.00000 | P[ 4] = 1.00000 | P[ 4] = 1.00000 |
| mt&nDNA-algorithm0 | speciesdelimitation=1 0 2 | speciesdelimitation=1 0 10 | speciesdelimitation=1 0 20 | speciesdelimitation=1 0 2 | speciesdelimitation=1 0 10 | speciesdelimitation=1 0 20 |
|  | heredity= 1 1 4 | heredity= 1 1 4 | heredity= 1 1 4 | locusrate = 1 10.0 | locusrate = 1 10.0 | locusrate = 1 10.0 |
|  | P[ 4] = 1.00000 | P[ 4] = 1.00000 | P[ 4] = 1.00000 | P[ 4] = 1.00000 | P[ 4] = 1.00000 | P[ 4] = 1.00000 |
| mt&nDNA-algorithm1 | speciesdelimitation=1 1 1 0.5 | speciesdelimitation=1 1 1.5 1 | speciesdelimitation=1 1 2 2 | speciesdelimitation=1 1 1 0.5 | speciesdelimitation=1 1 1.5 1 | speciesdelimitation=1 1 2 2 |
|  | heredity= 1 1 4 | heredity= 1 1 4 | heredity= 1 1 4 | locusrate = 1 10.0 | locusrate = 1 10.0 | locusrate = 1 10.0 |
|  | P[ 4] = 1.00000 | P[ 3] = 0.00320 | P[ 1] = 0.04205 | P[ 4] = 1.00000 | P[ 4] = 1.00000 | P[ 4] = 1.00000 |
|  |  | P[ 4] = 0.99680 | P[ 3] = 0.00080 |  |  |  |
|  |  |  | P[ 4] = 0.95705 |  |  |  |
| (b) ~ G (1, 10) and τ ~ G (2, 2000) | | | | | | |
| nDNA-algorithm0 | speciesdelimitation=1 0 2 | speciesdelimitation=1 0 10 | speciesdelimitation=1 0 20 | speciesdelimitation=1 0 2 | speciesdelimitation=1 0 10 | speciesdelimitation=1 0 20 |
|  | heredity= 1 4 4 | heredity= 1 4 4 | heredity= 1 4 4 | locusrate = 1 10.0 | locusrate = 1 10.0 | locusrate = 1 10.0 |
|  | P[ 4] = 1.00000 | P[ 4] = 1.00000 | P[ 4] = 1.00000 | P[ 4] = 1.00000 | P[ 4] = 1.00000 | P[ 4] = 1.00000 |
| nDNA-algorithm1 | speciesdelimitation=1 1 1 0.5 | speciesdelimitation=1 1 1.5 1 | speciesdelimitation=1 1 2 2 | speciesdelimitation=1 1 1 0.5 | speciesdelimitation=1 1 1.5 1 | speciesdelimitation=1 1 2 2 |
|  | heredity= 1 4 4 | heredity= 1 4 4 | heredity= 1 4 4 | locusrate = 1 10.0 | locusrate = 1 10.0 | locusrate = 1 10.0 |
|  | P[ 4] = 1.00000 | P[ 4] = 1.00000 | P[ 4] = 1.00000 | P[ 3] = 0.00035 | P[ 4] = 1.00000 | P[ 4] = 1.00000 |
|  |  |  |  | P[ 4] = 0.99965 |  |  |
| mt&nDNA-algorithm0 | speciesdelimitation=1 0 2 | speciesdelimitation=1 0 10 | speciesdelimitation=1 0 20 | speciesdelimitation=1 0 2 | speciesdelimitation=1 0 10 | speciesdelimitation=1 0 20 |
|  | heredity= 1 1 4 | heredity= 1 1 4 | heredity= 1 1 4 | locusrate = 1 10.0 | locusrate = 1 10.0 | locusrate = 1 10.0 |
|  | P[ 4] = 1.00000 | P[ 3] = 0.00030 | P[ 4] = 1.00000 | P[ 4] = 1.00000 | P[ 4] = 1.00000 | P[ 4] = 1.00000 |
|  |  | P[ 4] = 0.99970 |  |  |  |  |
| mt&nDNA-algorithm1 | speciesdelimitation=1 1 1 0.5 | speciesdelimitation=1 1 1.5 1 | speciesdelimitation=1 1 2 2 | speciesdelimitation=1 1 1 0.5 | speciesdelimitation=1 1 1.5 1 | speciesdelimitation=1 1 2 2 |
|  | heredity= 1 1 4 | heredity= 1 1 4 | heredity= 1 1 4 | locusrate = 1 10.0 | locusrate = 1 10.0 | locusrate = 1 10.0 |
|  | P[ 4] = 1.00000 | P[ 3] = 0.00015 | P[ 4] = 1.00000 | P[ 4] = 1.00000 | P[ 4] = 1.00000 | P[ 3] = 0.00030 |
|  |  | P[ 4] = 0.99985 |  |  |  | P[ 4] = 0.99970 |
