# Supplementary material for: Molecular phylogeny and taxonomy of the genus Vernaya (Mammalia: Rodentia: Muridae) with the description of two new species
Source: Ecol Evol. 2023 Nov 9;13(11):e10628. doi: 10.1002/ece3.10628 (PMC10636494; doi:10.1002/ece3.10628)
Supplement: Supplementary file 6 — Table S5. [file ECE3-13-e10628-s004.docx]

Supplementary table 5. Results of canonical discriminant analysis of the genus of *Vernaya*

|  |  | *V. foramena* | *V. fulva* | *V. meiguites* | *V. nushanensis* | Total |
| --- | --- | --- | --- | --- | --- | --- |
| Original counts | *V. foramena* | 7 | 1 | 0 | 0 | 8 |
|  | *V. fulva* | 1 | 8 | 0 | 0 | 9 |
|  | *V. meiguites* | 0 | 0 | 6 | 0 | 6 |
|  | *V. nushanensis* | 0 | 0 | 0 | 2 | 2 |
| Total |  | 8 | 9 | 6 | 2 | 25 |
| Percent ( % ) | *V. foramena* | 88 | 0 | 12 | 0 | 100 |
|  | *V. fulva* | 11 | 89 | 0 | 0 | 100 |
|  | *V. meiguites* | 0 | 0 | 100 | 0 | 100 |
|  | *V. nushanensis* | 0 | 0 | 0 | 100 | 100 |
